# The Promotion Effect of Low-Molecular Hydroxyl Compounds on the Nano-Photoelectrocatalytic Degradation of Fulvic Acid and Mechanism

**DOI:** 10.1007/s40820-016-0091-7

**Published:** 2016-05-04

**Authors:** Yifan Dong, Jinhua Li, Xuejin Li, Baoxue Zhou

**Affiliations:** 1 School of Environmental Science and Engineering, Shanghai Jiao Tong University, Shanghai, 200240 People’s Republic of China; 2 Key Laboratory for Thin Film and Microfabrication of the Ministry of Education, Shanghai Jiao Tong University, Shanghai, 200240 People’s Republic of China

**Keywords:** Fulvic acid, Nano-photoelectrocatalytic degradation, Promotion effect, Low-molecular hydroxyl compounds

## Abstract

**Abstract:**

A significant promotion effect of low-molecular hydroxyl compounds (LMHCs) was found in the nano-photoelectrocatalytic (NPEC) degradation of fulvic acid (FA), which is a typical kind of humic acid existing widely in natural water bodies, and its influence mechanism was proposed. A TiO_2_ nanotube arrays (TNAs) material is served as the photoanode. Methanol, ethanediol, and glycerol were chosen as the representative of LMHCs in this study. The adsorption performance of organics on the surface of TNAs was investigated by using the instantaneous photocurrent value. The adsorption constants of FA, methanol, ethanediol, and glycerol were 43.44, 19.32, 7.00, and 1.30, respectively, which indicates that FA has the strongest adsorption property. The degradation performance of these organics and their mixture were observed in a thin-layer reactor. It shows that FA could hardly achieve exhausted mineralization alone, while LMHCs could be easily oxidized completely in the same condition. The degradation degree of FA, which is added LMHCs, improves significantly and the best promotion effect is achieved by glycerol. The promotion effect of LMHCs in the degradation of FA could be contributed to the formation of a tremendous amount of hydroxyl radicals in the NPEC process. The hydroxyl radicals could facilitate the complete degradation of both FA and its intermediate products. Among the chosen LMHCs, glycerol molecule which has three hydroxyls could generate the most hydroxyl radicals and contribute the best effective promotion. This work provides a new way to promote the NPEC degradation of FA and a direction to remove humus from polluted water.

**Graphical Abstract:**

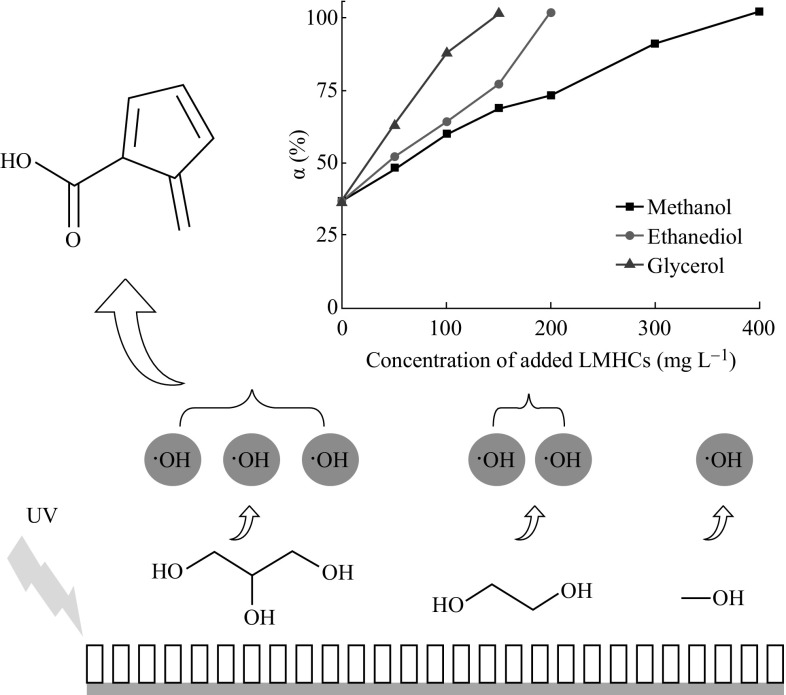

## Introduction

Fulvic acid (FA) is the main component of humus, which is a dominating natural organic matter (NOM) in the water body [1, 2]. Meanwhile, it is a refractory organic and also a major precursor of disinfection by-products (DBPs) which is formed in the process of chlorine disinfection in drinking water treatment. The DBPs are categorized as a kind of harmful materials which are carcinogenic, teratogenic, and mutagenic [3]. As a consequence, discovering a highly efficient and stable method to degrade FA is particularly important.

To remove FA from the water body, limited methods are found, which are enhanced coagulation, granular-activated carbon adsorption, and membrane filtration. Among them, the enhanced coagulation is the best way to control the amount of NOM. However, it is only effective to the mixture which includes large concentration of colloid humic acid and ineffective to the solution which has solute low molecular like FA in it [4, 5].

In recent years, to degrade a series of refractory organics, FA especially, the advanced oxidation technologies have become a research’s hot spot, because it can generate hydroxyl radicals which have well-degradation ability. As an effective advanced oxidation technology, the photoelectrocatalytic (PEC) technology has many advantages, such as good oxidation ability, high organic matter removal efficiency, and no secondary pollution and so on. TiO_2_ nanotube arrays (TNAs) are chosen to be a photoanode material, which has larger specific surface area and more stable structure than other forms of TiO_2_ [6–9]. Meanwhile, it shows better catalytic properties and could degrade more kinds of refractory organic matter [10–17].

The PEC degradation of organics could not only be affected by the type of photocatalyst, the configuration of reactor, but also be affected by the reaction medium [18–21]. For example, some chemical substances, such as tertiary butyl alcohol, phosphate, and carbonate could inhibit the hydroxyl radical’s activity, so they could further inhibit the PEC degradation of organics [22].

The feasibility of PEC technology used in the degradation of FA is proved in the limited previous researches [8, 9]. However, the reported method of FA degradation is inefficient and time-consuming, or the preparation of the catalytic material is very complex. In this work, in order to fast and accurately measure the degradation of organics and explore the degradation mechanism, an efficient catalytic material, TNAs, and a thin-layer PEC reactor were used. The PEC degradation of FA is under study, and a significant PEC promotion effect was found in the degradation of FA by adding low-molecular hydroxyl compounds (LMHCs) including methanol, ethanediol, and glycerol, which are widely existed in the water body and polluted water. The influence mechanism was proposed.

## Experiment

### Material and Sample Preparation

Unless specially indicated, all the reagents were analytical pure grade and were purchased from Sinopharm Chemical Reagent Company (Shanghai, China). All solutions were made up with high-purity de-ionized water (18 MΩ) purified by a Milli-Q purification system (Millipore Corporation, Billerica, MA), and the supporting electrolyte in the samples is a NaNO_3_ solution.

### Preparation of the TNA Electrode

The TiO_2_ nanotube electrodes were prepared according to the electrochemical anodic oxidation method which is reported in previous work [23]. The anode was titanium and the cathode was platinum. They were put into a mixture consisted of 1 mol L^−1^ NaF, 1 mol L^−1^ NaHSO_4_, 0.2 mol L^−1^ trisodium citrate, and NaOH was added to adjust the pH. The TiO_2_ nanotube electrodes would be formed in the solution under constant stirring for 6 h with an applied bias of 20 V. Then, they were annealed in a laboratory muffle furnace at 500 °C lasting for 3 h to form anatase TNAs.

### The Characterization Method of TNA Electrode

The surface morphology of TNAs working electrode was investigated by field emission scanning electron microscopy (Nova NanoSEM 450, FEI Company, USA) under a voltage of 5 kV. The crystalline phase of TNAs working electrode was identified by X-ray diffraction (XRD-6100, Shimadzu, Japan), using Cu *K*α (*λ* > 0.15406 nm) radiation at 40 kV and 30 mA at a scanning rate of 10° min^−1^ in the 2θ range from 10° to 90° at room temperature. The elements present in the TNAs electrode were identified by X-ray photoelectron spectroscopy (XPS, VG Microlab 310F, Al Ka radiation).

To explore this TNA’s photoelectric properties, the characterization of TNAs working electrode was investigated and the electrolyte solution was oxidized under three degradation conditions: photocatalytic (PC), electrocatalytic (EC), and photoelectrocatalytic (PEC). The PEC condition was performed by UV illumination from a 365 nm ultraviolet LED with a 37.4 mW cm^−2^ light intensity as well as a 2 V bias voltage, which avoid the photoinduced electron and the hole from recombining. The PC and EC condition were performed without bias voltage or UV illumination, respectively.

### Reactor Used in the Experiment

**Fig. 1:**
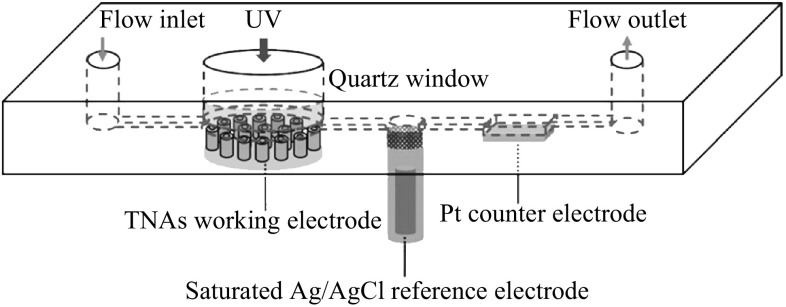
The structure of the thin-layer reactor

In this study, the reaction and degradation process were carried out in a thin-layer reactor. As shown in Fig. 1, the thin-layer reactor is a three-electrode system made up with six main sections: the TNAs working electrode, the platinum counter electrode, the saturated Ag/AgCl reference electrode, the flow inlet, outlet, and a quartz window with a diameter of 1 cm. Two polytetrafluoroethylene planks were adhered together to build a reaction cell. The thickness of the cell was controlled at most 0.1 mm to shorten the time of degradation and the distance of the mass transfer from the solution to the surface of electrode, and ensure the light transmittance of a 365 nm ultraviolet LED in the meanwhile. The potential and the current of the working electrode were controlled and monitored by an electrochemical workstation (CHI 610D, Chenghua, Shanghai) which was connected to the computer to record the photocurrent response signals.

### The Concentration Unit of the Organics and the Degradation Degree

The concentration unit of organics in oxygen equivalent (mg L^−1^) was used in this study, since the transfer of each 4 mol electrons in the PEC degradation process is equivalent to the consumption of 1 mol O_2_. This way will facilitate the evaluation of chemical reaction behavior of different organics as it loses the same amount of electrons [24].

The degradation degree (*α*) could be calculated according to Eq. 1, in which *Q*_net_ was the measured value of the charge transfer quantity and *Q*_th_ was the theoretical value of the charge transfer quantity in the PEC degradation process.

1 $$\alpha \, = Q_{\text{net}} /Q_{\text{th}}$$

**Fig. 2:**
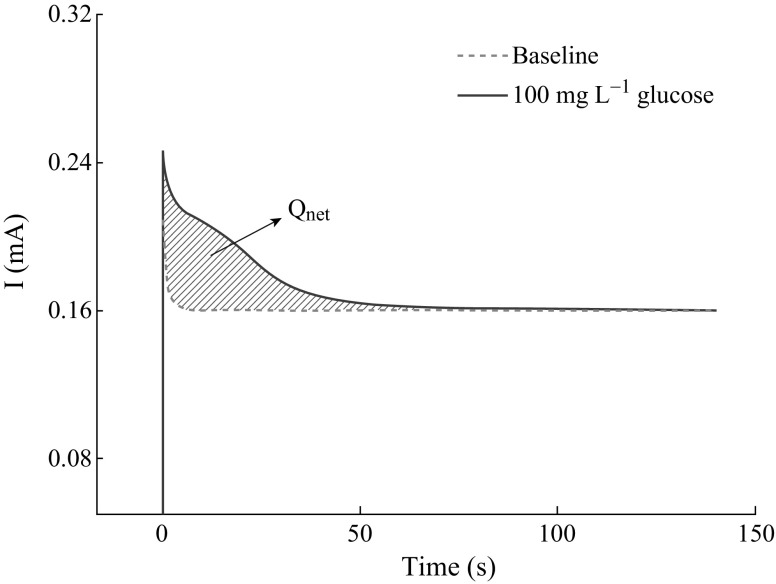
The response signals in the glucose degradation

*Q*_net_ could be obtained by computing the integral area of the response signals shown in Fig. 2 according to Eq. 2.

2 $$Q_{{{\mathbf{net}}}} = \, \int I{\text{dt}}$$

*Q*_th_ could be obtained by an exhausted PEC oxidation process of a standard substance, such as glucose [24, 25].

## Results and Discussion

### Characterization of TNA Electrode

**Fig. 3:**
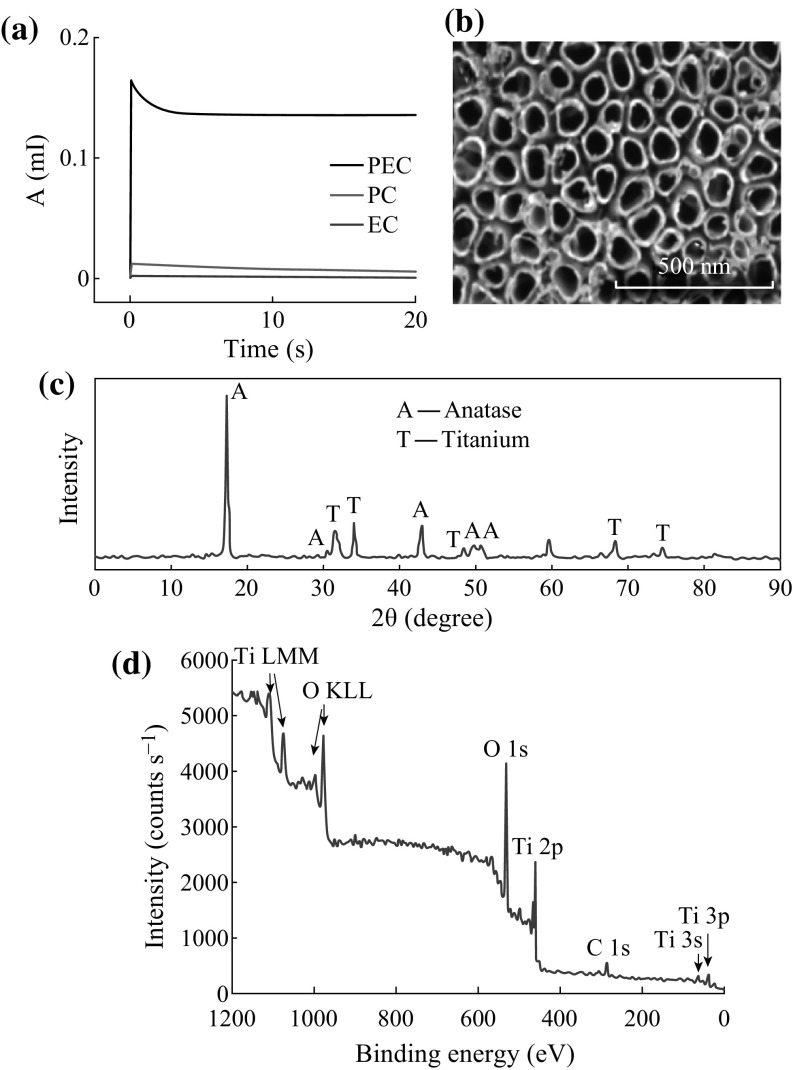
The characterization of TNA electrode. **a** photoelectric property, **b** SEM, **c** XRD pattern, and **d** XPS of TNA electrode

The *I*–*t* curves obtained from three degradation condition are shown in Fig. 3a. Both the current values of the PC and EC degradation are much smaller than the PEC degradation, so the degradations under PC and EC are ineffective, which illustrates the potential and UV light illumination work out a synergistic effect in the PEC oxidation process of the electrolyte solution (and as well as other matters, such as organics especially). The SEM of the TNAs shown in Fig. 3b reveals that the nanotubes are highly ordered. The XRD patterns of TNAs electrode shown in Fig. 3c indicate that the sample possesses characteristic peaks for the anatase phase. In order to further investigate the chemical composition and oxidation state of TiO_2_ nanotube arrays, XPS measurements were performed. Figure 3d shows a typical XPS survey scan for the TNAs material over a large energy range at low resolution, which represents Ti^4+^, O^2−^ of TiO_2_ and some traces of carbon. These results further confirm the successful preparation of TNAs.

### The Adsorption Property

**Fig. 4:**
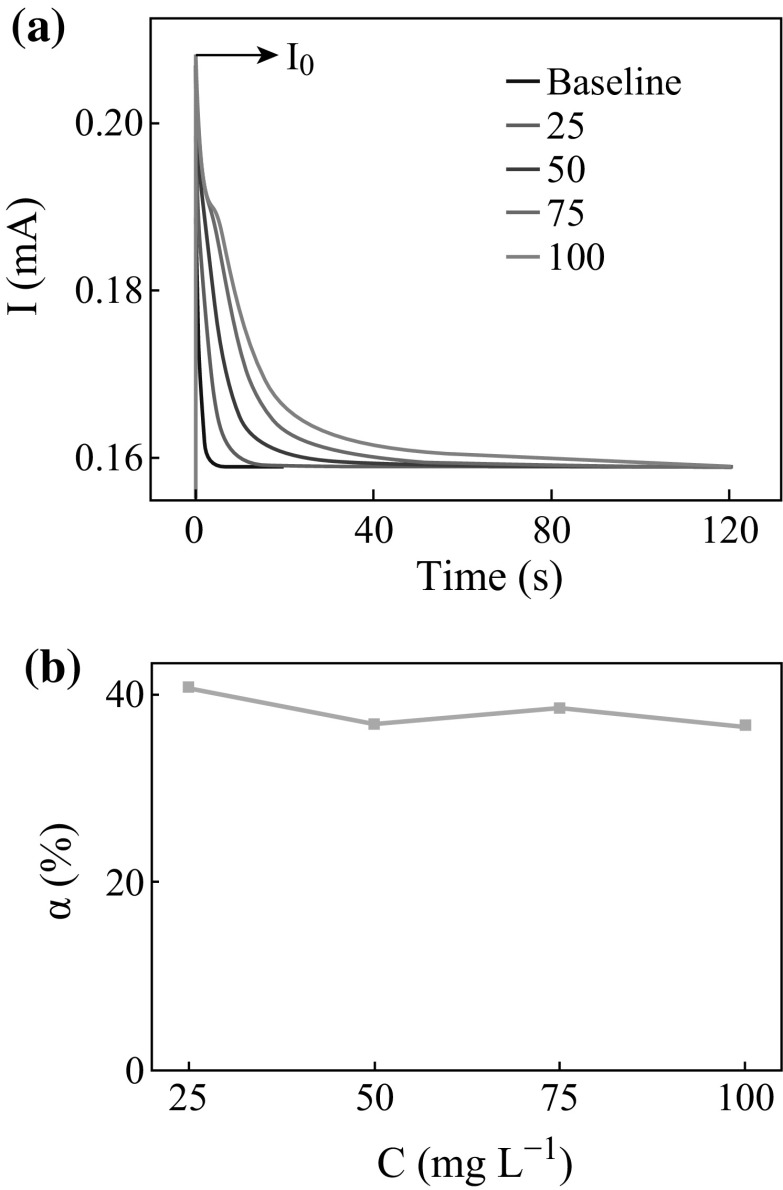
**a** The response signals, and **b** the degree of the degradation of FA in different concentrations

As shown in Fig. 4a, in the beginning of the FA’s PEC degradation, the initial instantaneous current value (*I*_0_) rises as the concentration of FA increases, which is related with its adsorption property [26].

According to the computer simulation, the relationship between the *I*_0_ and the concentration of FA could fit with the following equation:

3 $$y = { 6}. 7 6\,\times \, 10^{ - 5} \times { 43}. 4 4x/ \, \left( { 1 { } + { 43}. 4 4x} \right) \, + { 3}. 3 9 \, \times \, 10^{ - 5}\,\left( {R^{ 2} = \, 0. 9 7} \right)$$

Equation 3 is similar to the Langmuir adsorption equation, which could appropriately describe the adsorption property of the solute on the interface of solid material in the solution [27, 28]:

4 $$y = A \times B \times x/ \, \left( { 1 { } + B \times x} \right) \, + C$$

In the equation, ***A*** is the Langmuir current response constant, ***B*** is the adsorption constant of organics on the interface, and ***C*** is the polarization current (A).

**Fig. 5:**
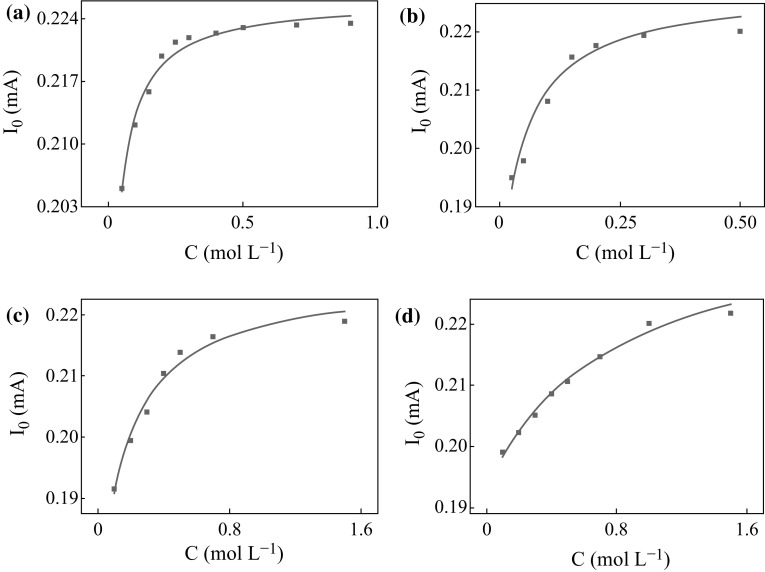
The Langmuir fitting curves of **a** FA, **b** methanol, **c** ethanediol, and **d** glycerol

From the result of fitting curve shown in Fig. 5a and the Langmuir adsorption equation, it is known that the adsorption constant of FA is 43.44. Based on the same method, the fitting curves of methanol, ethanediol, and glycerol shown in Fig. 5, could be obtained. Their adsorption constants were 19.32, 7.00, and 1.30, respectively. Thus, FA has the strongest adsorption property.

### The PEC Degradation Performance of FA

To explore the PEC degradation performance of FA, different concentrations of FA (25, 50, 75, and 100 mg L^−1^) were degraded in the thin-layer reactor. The response signals obtained from the above degradation are shown in Fig. 3a. According to Eqs. 1 and 2, the degradation degree (*α*) can be calculated, showing the range from 36 to 41 % as shown in Fig. 3b. Thus, FA could not be completely degraded alone in the PEC degradation.

The shapes of the *I*–*t* curve in Fig. 3a are similar. Before the degradation reaction, a portion of FA molecular could be adsorbed on the surface of the TNAs electrode when the solution was injected into the reactor because of its strong adsorption property. Thus, the initial instantaneous current value is quite high, and it could rise as the concentration increases. Thereafter, the current value reduces rapidly and the degradation degree is less than 40 %. It can be deduced that the hydroxyl radicals, which can degrade FA, have been consumed mostly in the early stage of the reaction. This could result in the reduction of the reaction rate and a steady state in the end. For this reason, it is necessary to increase the amount of hydroxyl radicals which could make a complete PEC degradation of FA.

### The PEC Degradation Performance of Three LMHCs

**Fig. 6:**
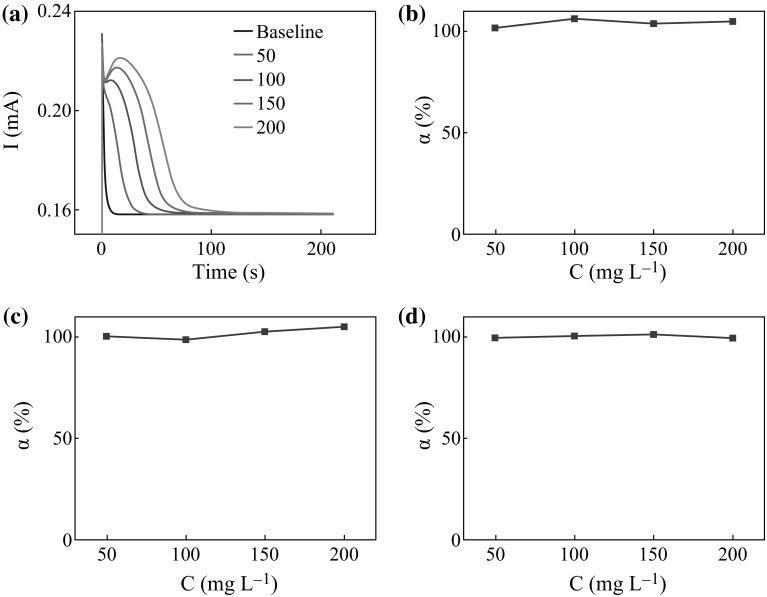
**a** The response signals of glycerol’s degradation. The degradation degree of three LMHCs in different concentrations: **b** glycerol, **c** methanol, and **d** ethanediol

To explore the influence of LMHCs, the PEC degradation performance of the LMHCs is investigated. The *I*–*t* curves in Fig. 6a show a similar shape by comparing the PEC degradation of glycerol in different concentrations. The current value declines a bit at the beginning, then increase for a short time (do not appear in low concentration, such as 50 mg L^−1^) and continuously declines until reaching a steady state. The increasing value represents the formation of the hydroxyl radicals from the degraded glycerol in the beginning of the PEC degradation, which could promote the degradation of the residual glycerol and the intermediate products. When most of glycerol is degraded, the promotion disappears and the current value reduces. According to Eq. 1, the degradation degree (*α*) of each concentration shown in Fig. 6b could be calculated. Obviously, the degradation degree of glycerol with different concentrations is all close to 100 %. The degradation degree of both methanol and ethanediol shown in Fig. 6c, d is also about 100 %. Thus, it can be said that the three LMHCs could be degraded completely.

### The Promotion Effect of LMHCs and the Mechanism

In this study, 50 mg L^−1^ FA is chosen as the target organics and the three LMHCs with the concentrations of 50, 100, 150, and 200 mg L^−1^ were added into the solution, respectively. Then the influence of each LMHC in the PEC degradation of FA was investigated.

**Fig. 7:**
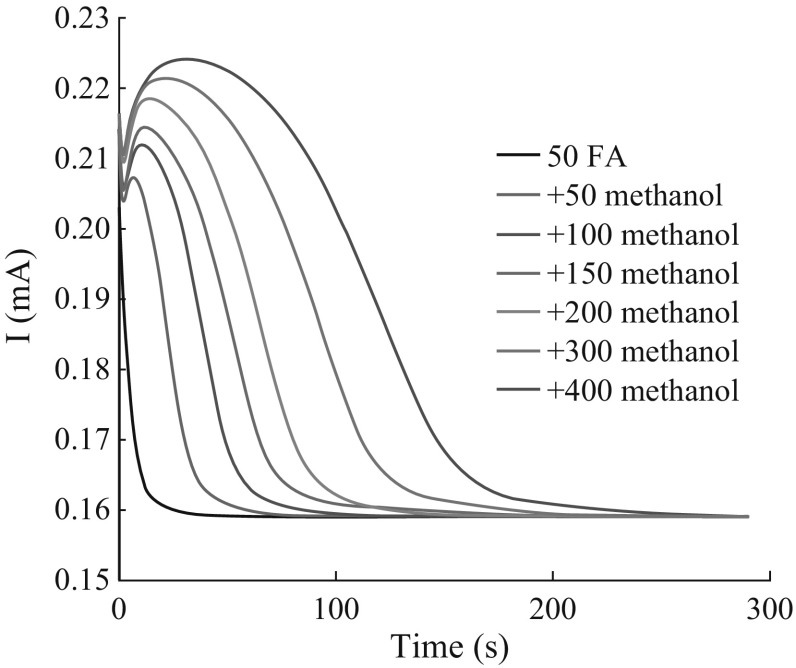
The response signals obtained from the degradation of FA which is added methanol

Figure 7 shows the *I*–*t* curves obtained from the PEC degradation of FA with addition of different concentrations of methanol. The integral area between two adjacent curves is equivalent to the increment of methanol and further degraded FA. The amount of charge transfer generated by the degradation of FA could be obtained by reducing the *Q*_th_ of added methanol from the *Q*_net_ of mixture calculated from the *I*–*t* curves according to the Eq. 2. According to the aforementioned, the three LMHCs could be degraded completely and their *Q*_th_ is represented by the quantity of transferred charges (*Q*_net-glucose_) generated in the exhausted PEC oxidation process of glucose in the same concentration. The each degradation degree of FA with addition of different concentrations of methanol could be calculated according to Eq. 1.

**Fig. 8:**
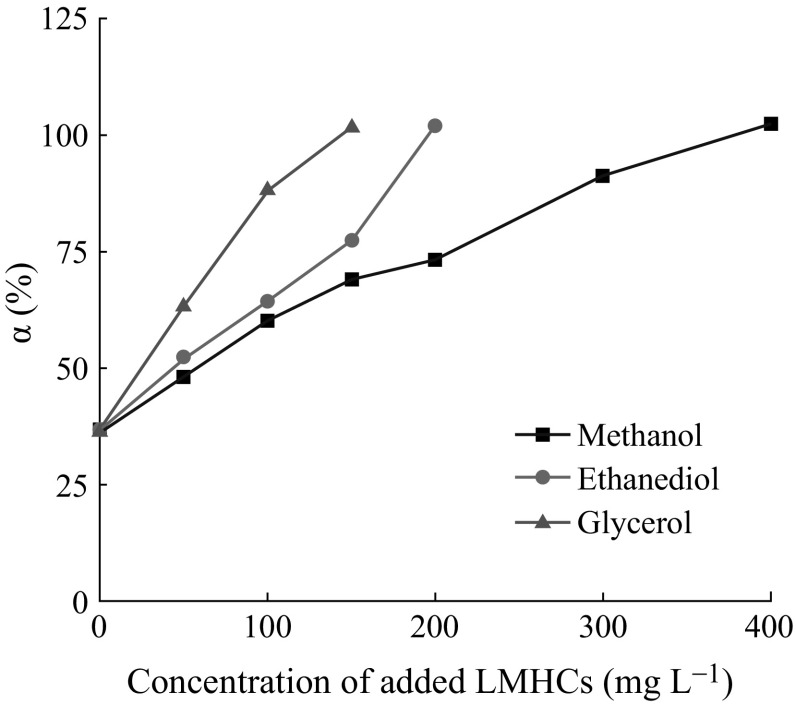
The degradation degree of FA added three LMHCs

The degradation degrees are shown in Fig. 8. As can be seen, methanol has an obvious promotion effect in the PEC degradation of FA. As the concentration of methanol increases, the degradation degree of FA rises. When 200 mg L^−1^ methanol was added, the degree is close to 80 %. After increasing the concentration of methanol, it could be found that almost 400 mg L^−1^ methanol is needed to make sure that 50 mg L^−1^ FA could be degraded completely. When different concentrations of ethanediol and glycerol are added, the degradation degrees of FA increase as well. Compared to the three LMHCs, glycerol shows the best promotion effect. To promote 50 mg L^−1^ FA degraded completely, only 100 mg L^−1^ glycerol is needed rather than 200 mg L^−1^ ethanediol and 400 mg L^−1^ methanol. Meanwhile, when 100 mg L^−1^ of glycerol is added, the degradation degree of 50 mg L^−1^ FA is much larger than the other conditions as shown in Table 1.

**Table 1:** The degree and the concentration of hydroxyl in each 100 mg L^−1^ LMHCs

100 mg L^−1^ LMHCs	Methanol	Ethanediol	Glycerol
Degree of degraded FA (%)	60.38	64.49	88.35
Hydroxyl concentration (mol L^−1^)	2.0833	2.5	2.6786

The mechanism of different LMHCs’ promotion effect can be attributed to the difference of the hydroxyl’s number in each molecule of the LMHCs. As shown in Table 1, the corresponding hydroxyl concentration that has 100 mg L^−1^ LMHCs influences the promotion effect. Obviously, glycerol provides the most hydroxyl radicals leading to the best promotion effect. Methanol molecule, which has only one hydroxyl, could not contribute enough hydroxyl radicals to promote the degradation. However, the difference of the adsorption constants could not influence their promotion effect. Although the adsorption of methanol is the highest, its promotion effect is not the best. It indicates that the amount of the hydroxyl in LMHCs makes a major effect.

## Conclusion

In this study, a significant promotion effect of LMHCs, including methanol, ethanediol, and glycerol, was found in the PEC degradation of FA. The degradation degree of FA alone is just about 40 %, as the concentration of FA ranged from 25 to 100 mg L^−1^. When LMHCs are added, the degree increases obviously. Meanwhile, the promotion effects of methanol, ethanediol, and glycerol increase successively. Their influence mechanism is that the hydroxyls own influence their promotion effects. The more hydroxyls LMHC has, the better promotion effect it makes. This work provides a new way to promote the PEC degradation of FA and a direction to remove humus from polluted water.
